# Analysis of Light Penetration Depth in Apple Tissues by Depth-Resolved Spatial-Frequency Domain Imaging

**DOI:** 10.3390/foods12091783

**Published:** 2023-04-25

**Authors:** Tongtong Zhou, Dong Hu, Dekai Qiu, Shengqi Yu, Yuping Huang, Zhizhong Sun, Xiaolin Sun, Guoquan Zhou, Tong Sun, Hehuan Peng

**Affiliations:** 1 College of Optical Mechanical and Electrical Engineering, Zhejiang A&F University, Hangzhou 311300, China; zhoutt@stu.zafu.edu.cn (T.Z.); 20180047@zafu.edu.cn (D.H.);; 2 College of Mechanical and Electronic Engineering, Nanjing Forestry University, Nanjing 210037, China; 3 College of Chemistry and Materials Engineering, Zhejiang A&F University, Hangzhou 311300, China

**Keywords:** light penetration depth, apple, spatial-frequency domain imaging, depth-resolved, bruise, scattering

## Abstract

Spatial-frequency domain imaging (SFDI) has been developed as an emerging modality for detecting early-stage bruises of fruits, such as apples, due to its unique advantage of a depth-resolved imaging feature. This paper presents theoretical and experimental analyses to determine the light penetration depth in apple tissues under spatially modulated illumination. Simulation and practical experiments were then carried out to explore the maximum light penetration depths in ‘Golden Delicious’ apples. Then, apple experiments for early-stage bruise detection using the estimated reduced scattering coefficient mapping were conducted to validate the results of light penetration depths. The results showed that the simulations produced comparable or a little larger light penetration depth in apple tissues (~2.2 mm) than the practical experiment (~1.8 mm or ~2.3 mm). Apple peel further decreased the light penetration depth due to the high absorption properties of pigment contents. Apple bruises located beneath the surface peel with the depth of about 0–1.2 mm could be effectively detected by the SFDI technique. This study, to our knowledge, made the first effort to investigate the light penetration depth in apple tissues by SFDI, which would provide useful information for enhanced detection of early-stage apple bruising by selecting the appropriate spatial frequency.

## 1. Introduction

**Figure 1:**
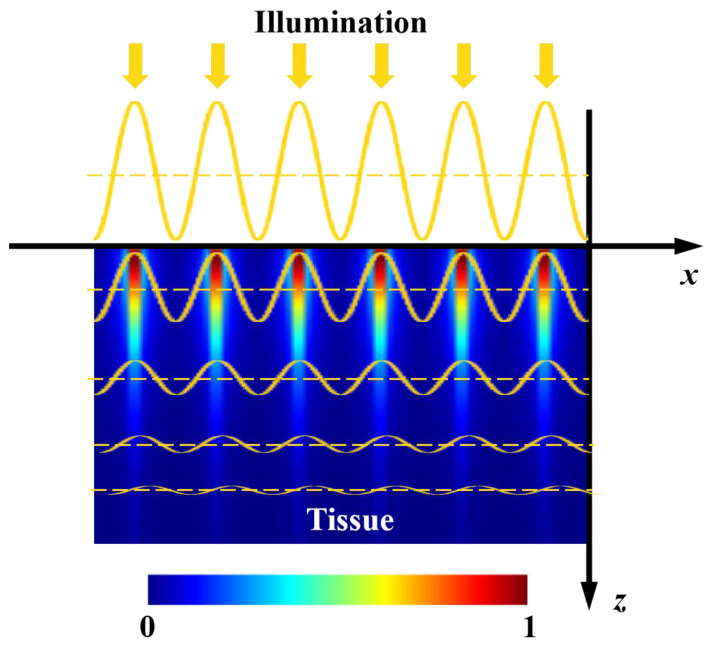
Schematic of light attenuation within a semi-infinite turbid medium under spatially modulated illumination.

Optical sensing techniques, such as near-infrared spectroscopy and hyperspectral imaging, have been extensively researched and increasingly utilized for detecting multiple defects of agro-food products [1,2,3]. The past decade has witnessed the development of spatial-frequency domain imaging (SFDI) for detecting various surface and subsurface defects of fruits [4,5,6,7]. As one of the typical defect types, surface bruises in apples often occur during harvest, transportation, storage, and sorting processes. Slight early-stage bruises are invisible to our naked eyes and are challenging to be recognized by traditional imaging techniques under uniform or diffuse illumination, which are more sensitive to the obvious surface properties. Thanks to frequency-dependent light attenuation within tissues, as depicted in Figure 1, SFDI enables acquiring depth-resolved information regarding tissue constituents and structure. Based on this remarkable feature, SFDI is proven to be capable of detecting the early-stage bruises of apples beneath the peels [6]. As opposed to the traditional uniform light imaging techniques, such as machine vision and hyperspectral imaging, spatially modulated light in a sinusoidal waveform is used in SFDI to acquire pattern images from samples. Through image demodulation and inverse estimation processing, SFDI produces 2-D optical property mappings in a pixel-by-pixel fashion, i.e., absorption coefficient ($\mu_{a}$) mapping and reduced scattering coefficient (${\mu^{\prime }}_{s}$) mapping. The differences of optical properties between non-bruised apple tissues and bruised ones can be directly used for early-stage bruise detection. It is well known that there are quite a lot of optical property measuring methods, e.g., time-resolved, spatially resolved, and integrating sphere, which have been employed for measuring optical properties of diverse agro-food products [8,9,10,11]. However, they are generally limited to point measurement and cannot attain depth-resolved information, resulting in great challenges in the nondestructive detection of early-stage bruises of apples [12,13]. In the technique of SFDI under the spatially modulated illumination, high-frequency light is more sensitive to the shallower tissue, while the low-frequency component has a much larger light penetration depth (~mm) [14,15], which provides a theoretical basis for detecting early-stage bruises of apples.

Knowledge of light penetration depth sampled by SFDI is of high significance for clinical and preclinical applications in the field of biomedicine [16,17,18]. For instance, the thickness of burned skin dictates the treatment protocol, highlighting the importance of understanding the detection depth in skin tissue [19]. SFDI has also been explored in deep-tissue applications, where it is essential to understand the penetration of collected photons in order to evaluate the maximum depth of measurable tumor contrast [20]. However, measuring light penetration depths in the field of biomedicine is not without limitations. The experiments generally require patients to remain completely still during a potentially long acquisition time in order to acquire full area scans. The exploration of the light penetration depth often requires prior knowledge about tissue optical properties that may not be valid in damaged tissue. Similar to that in the field of agricultural and food engineering, knowledge of light penetration depth is critical for enhancing the detection performance of early-stage bruises in apples by SFDI. Despite great progress being made for the bruise detection of apples, there still is a lack of exploration of quantifying light penetration depth in apple tissue. As mentioned above, SFDI has a remarkable advantage in subsurface (early-stage) bruise detection of apples, but the light penetration depth is reported to be limited in mm, implying that some internal bruises located in the region of deep tissue cannot be detected. It is thus desirable and also necessary to quantify light penetration depths under varying-frequency spatially modulated illumination, so as to better explore the potential of SFDI for early-stage bruise detection of apples in different depths, as well as to assess the severity of apple bruising. Up to now, the maximum detection depth in apple tissue using SFDI has been uncertain and there are few research studies focusing on studying light penetration depth. Lu and Lu [21] reported that the maximum light penetration depth was confirmed to be no more than three sheets of blank printing paper (or less than 400 μm). Their study investigated the light penetration depth from the aspect of demodulated images, which is different from our research in optical property estimation through inverse computation. Apple is taken as the experimental material in this study, which has different properties from the blank printing paper. The estimated optical property mappings could provide quantitative information in bruise detection (specific values of optical properties for non-bruised and bruised tissues), and thus further exploration of the light penetration depth could be implemented through these quantitative information.

Therefore, in this study, a set of well-designed experiments from theoretical simulation to practical implementation was performed to quantify the light penetration depth (especially for the maximum value) in apple tissue under spatially modulated illumination. The objectives were to (1) explore the light penetrating capacity of demodulated direct component (DC) and amplitude component (AC) images to prove our SFDI system performance; (2) conduct the simulation and practical experiments to investigate the light penetration depths in ‘Golden Cream Delicious’ apples with and without peels; and (3) validate the conclusion of the maximum light penetration depth in apple tissues by evaluating the performance of bruise detection.

## 2. Theoretical Formulation for SFDI

### 2.1. Image Formation and Image Processing

In SFDI, image formation involves two steps [22]: (1) the incident light interacts with the sample through absorption and multiple scattering, and (2) the light reemitted from the sample travels through a series of optical devices (e.g., lens, camera) of the imaging system, and eventually forms a digital image. As a general rule, SFDI is regarded as a linear, space-invariant technique which applies transfer function theory to an optical imaging system [23]. There are several factors negatively affecting the resolution and contrast of resulting images during image formation and processing, such as convolution operation and environmental noise. A mathematical method is used to analyze the image acquired by SFDI, which is generally composed of two parts. The first part is DC, i.e., $I_{DC}$ with the Fourier spectra centered at the origin; the other part is AC termed as $I_{AC}$, which is composed of an oscillatory or harmonic component, with the Fourier spectra shifted by positive or negative frequency ($f_{x}$ or $-f_{x}$) [24].

The process of optical property estimation from the remitted image in the SFDI can be roughly divided into two steps: acquisition of diffuse reflectance image through demodulation and estimation of optical property mapping through inverse computation. Due to the characteristics of high accuracy and easy implementation, phase shifting techniques are widely used for demodulation from sinusoidal fringe patterns. Three-phase demodulation (TPD) is a commonly used and effective method that uses three images with the phase offsets of −2π/3, 0, and 2π/3. Under the illumination of three phase-shifted sinusoidal patterns, the corresponding intensity images, i.e., $I_{1}\left(x,y\right)$, $I_{2}\left(x,y\right)$*,* and $I_{3}\left(x,y\right)$, can be expressed as follows [25]:

(1) $$I_{1}\left(x,y\right)=I_{DC}+I_{AC}\cos\left(2\pi f_{x}x-2\pi /3\right)$$

(2) $$I_{2}\left(x,y\right)=I_{DC}+I_{AC}\cos\left(2\pi f_{x}x\right)$$

(3) $$I_{3}\left(x,y\right)=I_{DC}+I_{AC}\cos\left(2\pi f_{x}x+2\pi /3\right)$$

where (x,y) represents the spatial coordinates, $f_{x}$ is the spatial frequency along the *x*-axis direction, and $I_{DC}$ and $I_{AC}$ are the direct and amplitude components, respectively. For the purpose of simplicity, we will drop off the coordinate notation. From Equations (1)–(3), the DC and AC images can be obtained by the following equations [15]:

(4) $$I_{DC}=\frac{1}{3}\left(I_{1}+I_{2}+I_{3}\right)$$

(5) $$I_{AC}=\frac{\sqrt{2}}{3}\sqrt{{\left(I_{1}-I_{2}\right)}^{2}+{\left(I_{1}-I_{3}\right)}^{2}+{\left(I_{2}-I_{3}\right)}^{2}}$$

### 2.2. Image Contrast

Light penetration features are of primary concern for the demodulated images, which are also critical for fruit bruise detection. To our knowledge, it is challenging to assess the light penetration capability, because it is largely dependent on tissue physicochemical properties and illumination conditions. For the bruised apples illuminated under spatially modulated illumination with varying frequencies, light penetration capability could essentially determine the thickness of the tissue that light passes through. In this study, we introduce image contrast and the ratio of peak to valley’s intensity (PVR) to evaluate the light penetration capability, which will be introduced in Section 3.2. Examination of the composition of photons backscattered from a turbid medium will provide qualitative insights into the relationship between light penetration depth and image contrast. The ballistic photons experience one or more backward and forward scattering events before exiting from the tissue. Due to the shortest traveling path, they suffer from minimal scattering and thus can deliver image information with superior resolution and contrast. However, the information generated by ballistic photons is more about the superficial layer of the medium in one mean free path (MFP) [26], around 100 μm for fruit tissue such as apple (assuming the value of ${\mu^{\prime }}_{s}$ is equal to or larger than 1.00 mm^−1^). The weakly scattered photons provide information on deeper, subsurface tissues, and they are still capable of forming well-resolved images due to limited scattering events. In summary, the tradeoff should be carefully considered between the light penetration depth and image contrast, while selecting spatial frequency in SFDI [26,27].

The $I_{DC}$, which contains a larger contribution of diffusive photons, probes a deeper region of sample tissues than $I_{AC}$, while $I_{AC}$ contains more ballistic and weakly scattered photons, resulting in better image contrast [15]. High-frequency illumination is more likely to enhance image contrast. Presented in the following sections are well-designed experiments to quantitatively determine the relationship between image contrast and the depth-resolved imaging feature of SFDI.

### 2.3. Light Penetration

In diffuse optics, the light penetration depth δ in biological tissues can be attained from the response to an infinitely narrow photon beam normally incident on a semi-infinite medium. For the case where that photon’s propagation depth z is larger than the light penetration depth, internal fluence distribution predicted from diffusion theory should be [28]:

(6) $$\emptyset \left(z\right)=\emptyset_{0}\;k\;\exp\left(-z/\delta \right)$$

where *k* is a scalar that depends on the amount of backscattered reflectance, $\emptyset_{0}$ is the incident irradiance, and ∅(z) represents a function of photon fluence. The light penetration depth is defined as [29]:

(7) $$\delta =\frac{1}{\sqrt{3\mu_{a}\left(\mu_{a}+\mu_{s}\left(1-g\right)\right)}}=\frac{1}{\mu_{eff}}$$

where $\mu_{a}$ is the absorption coefficient, $\mu_{s}$ is the scattering coefficient, *g* is anisotropy factor, and $\mu_{eff}$ is the effective attenuation coefficient.

According to this, the light penetration depth is estimated to be 1.50–6.00 mm for apple tissues with typical $\mu_{a}$ and $\mu_{s}$ coefficients of 0.01–0.05 mm^−1^ and 9.00–28.00 mm^−1^, respectively. However, the estimated depth does not always stand for the actually detectable depth for a general imaging system under spatially extended wide-field or broad-beam illumination. The fluence rate and reflectance properties of spatially modulated photon density plane waves in the SFDI are described in the study of Cuccia, Bevilacqua, Durkin, Ayers, and Tromberg [22], in which the effective penetration depth ${\delta^{\prime }}_{eff}$ is concisely defined as:

(8) $${\mu^{\prime }}_{eff}={\left(\mu_{eff}^{2}+k_{x}^{2}+k_{y}^{2}\right)}^{1/2}=\frac{1}{{\delta^{\prime }}_{eff}}$$

where ${\mu^{\prime }}_{eff}$ is a scalar attenuation coefficient, $k_{x}$ and $k_{y}$ are variable coefficients related to spatial frequencies $f_{x}$ and $f_{y}$ ($k_{x}$ = $2\pi f_{x}$, $k_{y}$ = $2\pi f_{y}$), and ${\delta^{\prime }}_{eff}$ is the effective penetration depth, which is inversely proportional to spatial frequency. The above mathematical formula just provides a simple conceptual framework to understand the transmission of modulated scalar photons in a turbid medium. In practice, the detected signal is mostly due to the photons backscattered close to the illumination source, which corresponds to a far more superficial depth of tissue interrogation than that derived from diffuse light attenuation [30]. This is equivalent to calculating the relative probability that a photon will visit a certain location in tissue before its detection. In the reflectance measurement geometry with spatially modulated illumination, the reemitted light intensity decays by many orders of magnitude within millimeters. Therefore, in using the formula to calculate the light penetration depth, there directly exists some unreasonable aspects. In this study, well-designed experiments were conducted, coupled with two evaluation parameters (image contrast and PVR), to explore the light penetration depth in apple tissues using SFDI.

## 3. Materials and Methods

### 3.1. SFDI System

**Figure 2:**
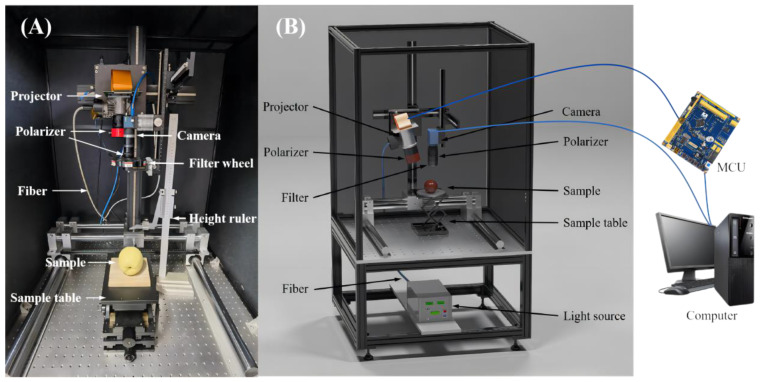
(**A**) Physical and (**B**) schematic maps of spatial-frequency domain imaging system.

An in-house assembled SFDI system, as illustrated in Figure 2, mainly consisted of a 150 W DC-regulated halogen fiber optic light source (Fiber-Lite DC950, Dolan-Jenner, Boxborough, MA, USA), a light-guide fiber (MSG4-2200S, MORITEX Corporation, Saitama, Japan), an 8-bit camera (MER-131-210U3M NIR, Daheng imaging vision Corporation, Shanghai, China) coupled with a C-mount zoom lens (HN-0816-5M-C2/3X, Daheng imaging vision Corporation, Shanghai, China) for vertically shining over a field of view (FOV, 11.5 × 11.5 cm^2^), a filter wheel (BOCIC Co., Ltd., Beijing, China) comprising six bandpass filters (550, 600, 630, 675, 710 and 730 nm), a microcontroller unit (MCU) (STM32F103ZET6, Opendv, Guangzhou, China), a three-axis manual displacement platform (THZ210, Runjia Pneumatic, Shenzhen, China) for holding samples, and an optical projector (DLi6500 1080p Optics Bundles, TI, Austin, TX, USA) for generating sinusoidal patterns. The projector was slightly angled at 12 degrees relative to the vertical axis to mitigate the image distortion, based on our tests and preliminary experiments, which was also confirmed by Lu et al. (2017) for constructing a multiple structured-illumination reflectance imaging system [31]. The MCU could take control of the projector for synchronous pattern projection and image acquisition with the camera. A pair of cross linear polarizers was mounted in front of the lens of the projector and camera to suppress specular reflectance from samples. The aforementioned components were mounted on an optical platform (SPL-R-0910, SPL-Tech, Hangzhou, China) and enclosed in a dark chamber for reducing the influence of external stray light.

### 3.2. Experiments

#### 3.2.1. Experiment 1: Penetrating Capacity of DC and AC Images

**Figure 3:**
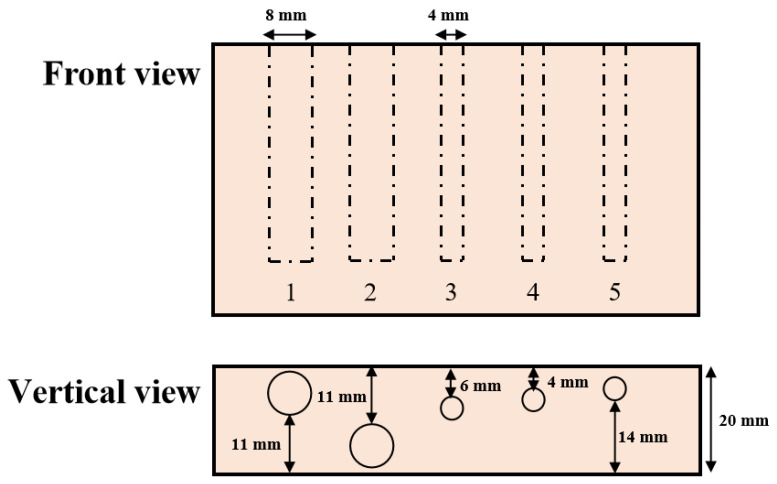
Schematic of relative positions and sizes of holes in the nylon sample filled with absorbing ink solution.

The first experiment was to verify the light-penetrating capacity of DC and AC images and thus prove our SFDI system performance. The experiment was conducted using a nylon slab with high scattering characteristics, which was drilled with five cylindrical holes running parallel with the surface. As shown in Figure 3, the five holes with the diameter of 4 mm or 8 mm were set up at the depths of 1 mm, 11 mm, 6 mm, 4 mm, and 2 mm, from left to right. Number the five holes in sequence as 1, 2, 3, 4 and 5. All the holes were filled with 100-times diluted India ink as absorbers for absorption property comparison with the bull material and then sealed with adhesive black tape. A sequence of sinusoidal patterns, covering 18 frequencies [0.01:0.01:0.15, 0.20, 0.25, 0.30] mm^−1^, was generated in Matlab R2020a (The Mathworks, Inc., Natick, MA, USA) for sample illumination. Three phase-shifted pattern images were acquired at each spatial frequency with the phase offsets of −2π/3, 0, and 2π/3, respectively. A standard whiteboard with the calibrated reflectance rate of 99% was imaged first under planar illumination to correct the non-uniformity of the source illumination [32]. Image demodulation was then used to generate the DC and AC images, according to Equations (4) and (5).

#### 3.2.2. Experiment 2: Investigating Light Penetration Depth

The second experiment was to quantify the light penetration depth in apple tissue under sinusoidal illumination. ‘Golden Cream Delicious’ apples, which were free of visual blemishes or defects and grown in Shandong, China, were purchased from a fruit market. Both simulation and practical experiments were carried out. In the first, the simulation methodology proposed by Hayakawa et al. [33] was adopted to roughly determine the optical sampling depth in apple tissues. In this method, Monte Carlo (MC), which has been widely applied to simulate light propagation in single- and multiple-layered biological tissues, was employed for the simulation experiment [8,29,34]. Optical property parameters ($\mu_{a},{\mu^{\prime }}_{s}$) of apple tissues at the six wavelengths were measured by our integrating sphere system [35], which were taken as the inputs in the simulation.

**Figure 4:**
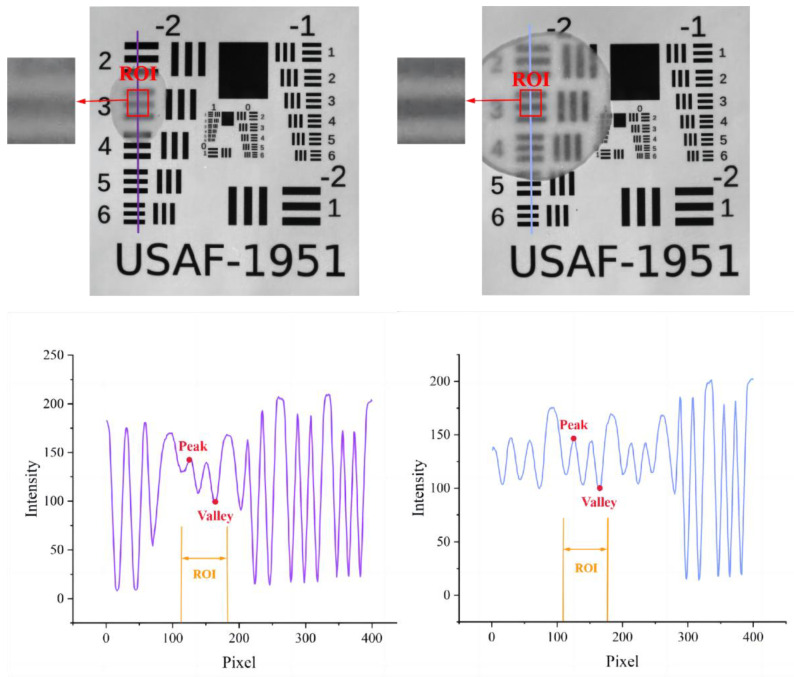
Example images of the USAF-1951 target covered with apple slices with peel (**left**) and without peel (**right**) for calculating image contrast (**up**) and PVR (**down**) in selected region of interest (ROI).

For the practical experiment for validating the results of the simulations, there were two types of samples, apple slices and a USAF-1951 target. A slicer was used to produce apple slices with different thicknesses with peel [0.9, 1.0–1.1, 1.3, 1.5–1.6, 1.6–1.7, 1.8, 2.1–2.2, 2.4–2.5, 2.6, 3.0, 3.4, 3.8, 4.0] mm and without peel [0.8, 1.0, 1.2–1.3, 1.5, 1.6, 1.8, 2.0, 2.3, 2.5–2.6, 2.7–2.8, 3.0, 3.2, 3.6, 4.0] mm. These varying-thickness apple slices were used to cover the USAF-1951 target, which was made of fiber material with high scattering properties, and their combination was imaged by the SFDI system, illuminated with a sequence of frequencies of 0.05, 0.10, 0.15, 0.20, 0.25, and 0.30 mm^−1^. It is apparent that the light has completely penetrated though the apple slice when the black horizontal bars of the USAF-1951 are recognized, as shown in the region of interest (ROI) in Figure 4. Two parameters in the ROI, i.e., image contrast and PVR, as mentioned above, were calculated to resolve the image details. Image contrast was evaluated based on the Michelson contrast metric (C_M_) [36]:

(9) $$C_{M}=\left(I_{max}-I_{min}\right)/\left(I_{max}+I_{min}\right)$$

where $I_{max}$ and $I_{min}$ denote the maximum and minimum intensities of the image in ROI, respectively. Another evaluating parameter, PVR, defined as the ratio of peak and valley’s intensity [37], was also determined from the captured images:

(10) $$PVR=I_{peak}/I_{valley}$$

where $I_{peak}$ and $I_{valley}$ denote the intensities of the peak and valley in the ROI, respectively.

#### 3.2.3. Experiment 3: Detecting Early Bruises in Apples

**Figure 5:**
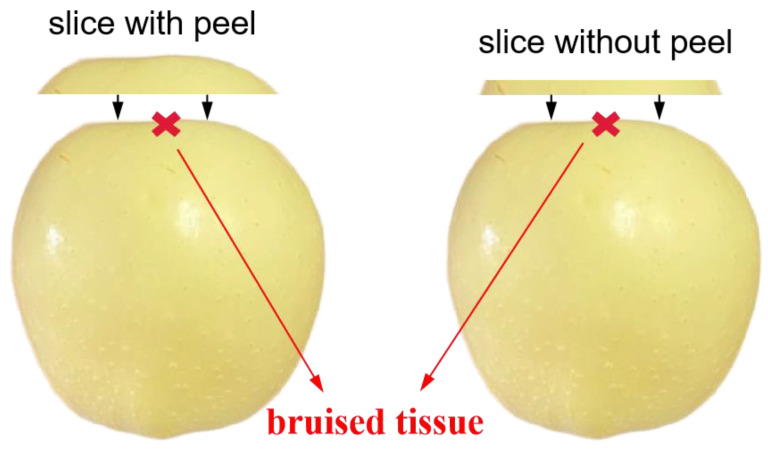
The diagram of peeled bruised apples covering with varying-thickness slices with (**left**) and without peels (**right**).

The third experiment was to test and verify the results of light penetration depths in apple tissues achieved from experiment 2. Impact tests were conducted to induce bruises in the apples with a wooden ball attached to one end. The wooden ball (6 cm in diameter and 105 g in weight) fell freely from the rest position at a certain height to impact the apple at its equatorial area. The peel on the surface of bruised tissue was cut off to eliminate its effect on detection performance. The used frequencies were the same as experiment 2. As shown in Figure 5, apple slices with and without peels in different thicknesses were used to cover the bruised tissues. Image demodulation and inverse parameter estimation were carried out to obtain the optical property ($\mu_{a}$ and ${\mu^{\prime }}_{s}$) mappings of the apples covered with slices. It is supposed that if the bruised tissue could be recognized in mappings, the light can penetrate through the apple slice, and the light penetration depth under this illumination should be equal to or larger than the thickness of the apple slice.

## 4. Results and Discussion

### 4.1. Penetrating Capacity of DC and AC Images

**Figure 6:**
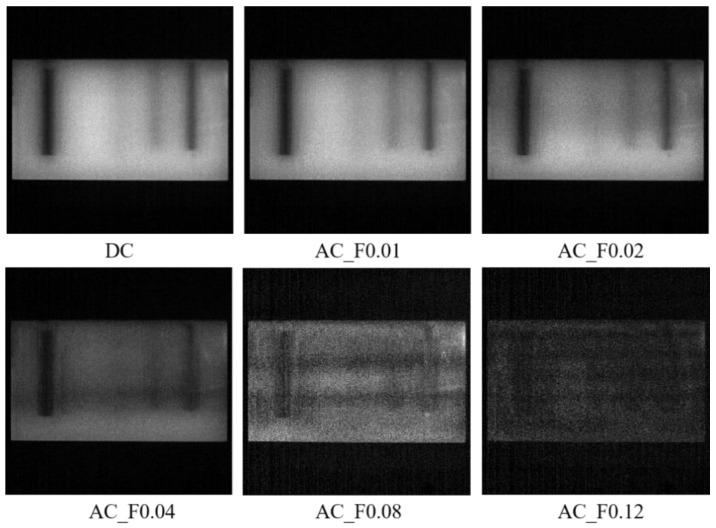
Demodulated DC (direct component) and AC (amplitude component) images of the nylon sample at a sequence of spatial frequencies of 0, 0.01, 0.02, 0.04, 0.08, and 0.12 mm^−1^, from (**top** **left**) to (**bottom** **right**).

Figure 6 shows the demodulated DC and AC images of the nylon sample. The filled India ink made the hidden holes relatively dark because of its strong light-absorbing capacity. In the DC image (equivalent to uniform illumination), the first hole was easily recognized but with invisibility on the top of the liquid column caused by an unwanted air bubble. The second hole was almost invisible because it was too deep (11 mm) from the tissue surface. For the three hidden holes on the right, the black liquid column became visually fuzzier along with larger distance from the surface. The AC image at the frequency of 0.01 mm^−1^ showed similar details to the DC image. As the frequency increased from 0.02 to 0.12 mm^−1^, the color of the leftmost column became more and more light, and the other columns revealed a reduction in the image invisibility. It was noticed that as the frequency increased, the surface texture of the nylon sample was revealed to a certain degree, with the images becoming less smooth. These observations implied that higher-frequency illumination brought about less light interrogation with deep tissues, resulting in a shallower light penetration depth. A similar finding was also reported in the previous study [15]. When the frequency reached 0.12 mm^−1^, all the holes became blurry due to insufficient light interrogation. Given all of that, the DC (0 mm^−1^) image had more light interrogation with deep tissues, while the AC images showed varying light penetration depths and image resolutions with different frequencies. It is suggested that the low-frequency component could penetrate deeper into the tissues than the high-frequency part, which is called the depth-resolved characteristic of SFDI in this study.

### 4.2. Investigating Light Penetration Depth

**Figure 7:**
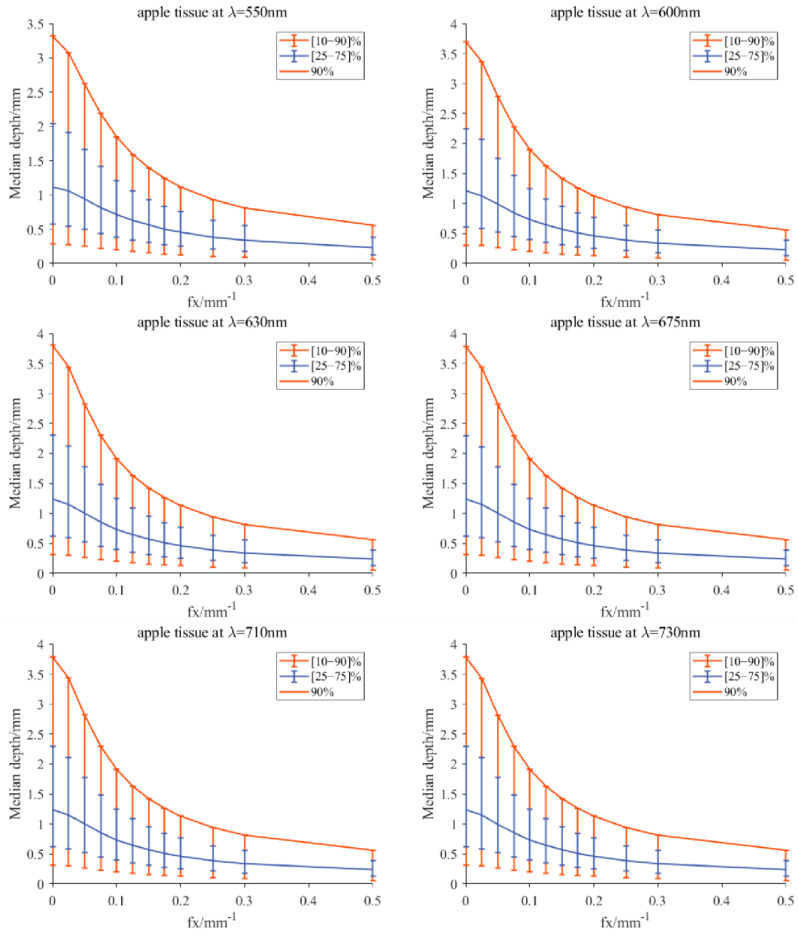
Simulated median optical detection depth for the apple tissue at six wavelengths, which is estimated using a scaled lookup table derived from MC simulations to the radiative transport equation in the spatial-frequency domain [33]. The median detection depth is the depth that encloses the photon trajectories responsible for 50% of the detected light, and accordingly, the vertical-capped lines in the figure correspond to detection depths responsible for 25% (**lower**) and 75% (**upper**) of the detected light.

Table 1 showed the measured values of optical property parameters ($\mu_{a},{\mu^{\prime }}_{s}$) of apple slices without peel using the integrating sphere technique. By inputting the optical property values manually into the developed program, the light penetration depth for the apple tissue could be simulated by consulting a scaled lookup table derived from MC simulations to the radiative transport equation in the spatial-frequency domain. Figure 7 displays the simulated results for the apple tissue at six wavelengths (550, 600, 630, 675, 710, and 730 nm), in which the median sampling depth with a [25–75]% fraction of the total measured diffuse reflectance was recognized as the critical metric for light penetration depth [33]. It was observed in the simulation results that light penetration depths increased slowly with the wavelengths, which is similar to the findings reported by Zhao et al. [17]. At 550 nm, the median sampling depth with [25–75]% was slightly smaller than that of the other five wavelengths, which was approximately in the range of 0.6–2.2 mm. It demonstrated that the 25–75% measured reflectance had the opportunity to interact with the tissue in the depth of 0.6–2.2 mm. Similarly, Lammertyn et al. [38] reported that the maximum light penetration depth in Jonagold apples at 692 nm was about 2 mm, which agrees well with the finding in this study. In the report of Binzoni et al. [39], the light propagation behavior is that the photons reaching the detectors do not go very deep and thus the information contained in the spectral images comes from a depth that does not exceed 2–3 mm. The above experimental results were all consistent with our simulation results. However, the light penetration depth (less than 400 μm) reported by Lu and Lu [21] was much smaller than the 2.2 mm. One potential reason is the evaluation level. Lu and Lu investigated the detection depth through demodulated images, while we studied the penetration depth from the aspect of optical property estimation. Furthermore, the custom-defined acceptable resolution and contrast would also affect the detection depth. Hence, it was concluded that the light penetration depths in apple tissues were close to each other at the six wavelengths, with values of no more than 2.2 mm.

**Figure 8:**
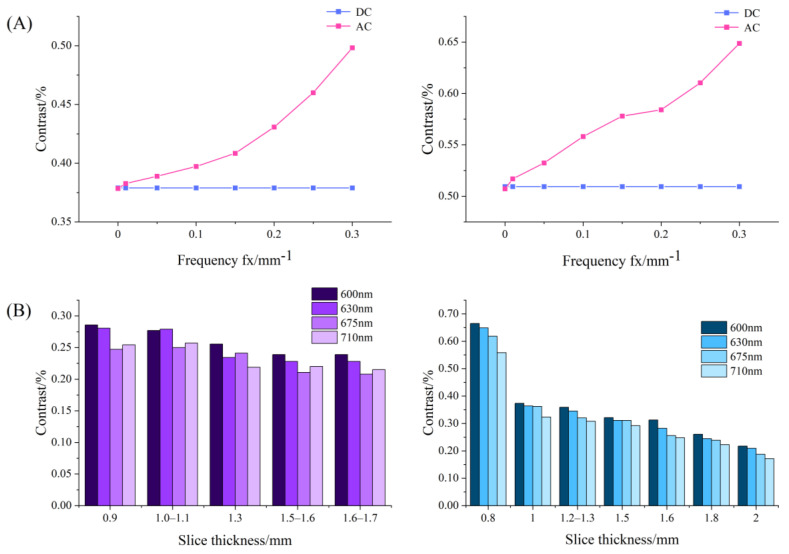
(**A**) Contrast variation with spatial frequencies for the direct component (DC) and amplitude component (AC) images of USAF-1951 target covered with apple slices with (left) and without peel (right); (**B**) histogram of contrast values for the DC images of USAF-1951 target covered with apple slice with (left) and without peel (right) in different thicknesses.

Figure 8A shows the contrast variation with frequencies for the DC and AC images of the USAF-1951 target covered with apple slices at 630 nm. For both the slices with and without peel, DC images gave almost a constant value of image contrast since they were independent of spatial frequency, while AC images showed much higher contrast values, which rose steadily with the spatial frequency. These findings indicated that AC images, which are unique to SFDI, enhanced image contrast compared to DC images, demonstrating that SFDI is superior to conventional uniform light imaging techniques in image contrast. Figure 8B shows the histogram results of image contrast for the DC images of the USAF-1951 target covered with apple slices at the wavelengths of 600, 630, 675, and 710 nm. It was noticed that the contrast values decreased with the thickness of apple tissue, as well as the wavelength. A special case occurs from 675 nm to 710 nm, in which there is a small rise of the contrast for some thicknesses. This is because the reflected signal intensity was generally poor in 630–690 nm due to strong absorption of chlorophyll. When removing the influence of the peel (right panel in Figure 8B), there was a steady decreasing trend for the image contrast with the wavelength, because the pigment had little effect on apple flesh tissue.

**Figure 9:**
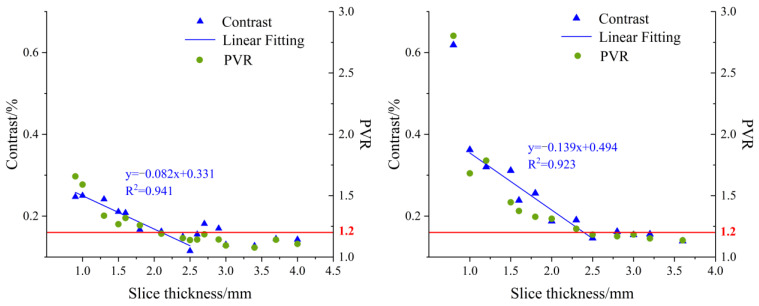
Contrast (left axis) and PVR (right axis) variation at 630 nm for direct component (DC) images of USAF-1951 target covered with different-thickness apple slices ((**left**) with peel; (**right**) without peel).

Figure 9 shows statistical results of contrast and PVR at 630 nm for the DC images of the USAF-1951 target covered with different-thickness apple slices. It was observed that the contrast values in the left chart decreased with the thickness of the apple slice. There was an approximate linear relation as the slice thickness ranged from 0.9 mm to 2.5 mm. A similar result was found when analyzing the image contrast in the right chart, with a narrow thickness range of 1.0–2.5 mm. It was noticed that the distribution of image contrast in the left chart (with peel) was more disperse and irregular than that in the right chart. This could be attributed to the influence of pores on the surface tissue of apple peel. On the other hand, PVR showed a gradual decreasing trend with the slice thicknesses. The black horizontal bars of the USAF-1951 target were hardly recognized while PVR was reduced to a certain value. In order to determine the light penetration depth in apple tissue, the threshold value of PVR in the slice thickness range of 0.9–2.5 mm, in which a linear relationship between the contrast and slice thickness was observed, was tested by large-scale experiments and finally set as 1.2. From this aspect, the light penetration depths in apple tissues with and without peels were determined as 0–1.8 mm and 0–2.3 mm, respectively. These results were quite similar to those obtained in the simulation experiments (0–2.2 mm). The differences between the simulation and practical experiments could be caused by many factors. For example, the apple optical properties, which were taken as the inputs in the simulations, are prone to measurement errors of the integrating sphere system, and thus lead to deviations for the simulation results. In addition, it is challenging to consider all the experiment details completely, such as the minute space between the covered apple slice and USAF-1951 target, and they may cause potential effects on our data analysis and final results.

### 4.3. Validation in Detecting Early-Stage Bruise of Apple

**Figure 10:**
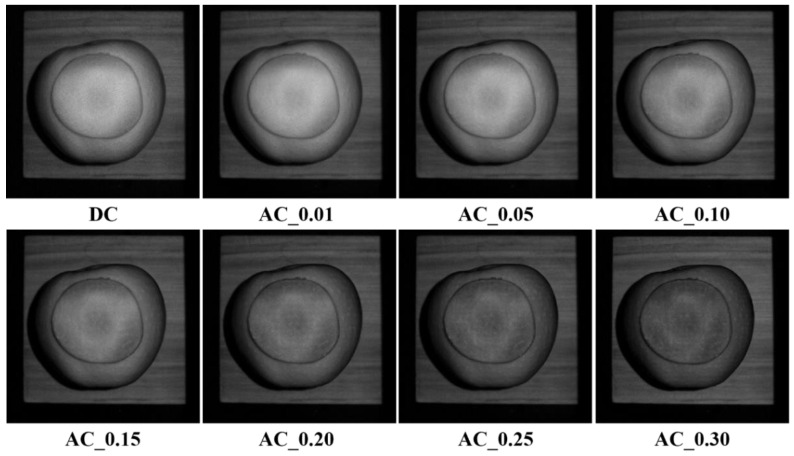
Demodulated images of the bruised apple covered with an 0.8 mm thick slice without peel at a sequence of spatial frequencies of 0, 0.01, 0.05, 0.10, 0.15, 0.20, 0.25, and 0.30 mm^−1^, from top left to bottom right.

Experiments on early-stage bruise detection of apples were conducted to validate the results of light penetration depth in apple tissues. The apple was peeled first to make the bruised tissue visible, and then it was covered with different-thickness apple slices with or without peels. Spatial-frequency domain images were acquired, followed with image demodulation and inverse estimation for generating optical property mappings. It was supposed that the bruised apple tissue could be detected if the light penetration depth was equal to or larger than the thickness of the covered apple slice. Figure 10 shows the demodulated images of the bruised apple covered with a 0.8 mm thick apple slice without peel. The AC images at certain spatial frequencies enhanced the bruised feature compared with the DC image. Strong contrast and surface texture variation were observed with the increased frequency. These findings indicated the enhanced capability of SFDI for detecting early-stage bruises of the apples, in comparison with the imaging techniques under uniform or diffuse illumination. It was believed that inverse parameter estimation for optical property mappings would provide more useful information for qualitative and quantitative analyses of bruise detection [32]. Therefore, the absorption and reduced scattering coefficient mappings were produced in our next step. The previous studies reported that bruising changed the optical properties of apples, particularly the reduced scattering coefficient, resulting in the difference between bruised and non-bruised tissues. The bruising detection based on the reduced scattering coefficient mapping was further analyzed to validate the results of light penetration depth in apples.

**Figure 11:**
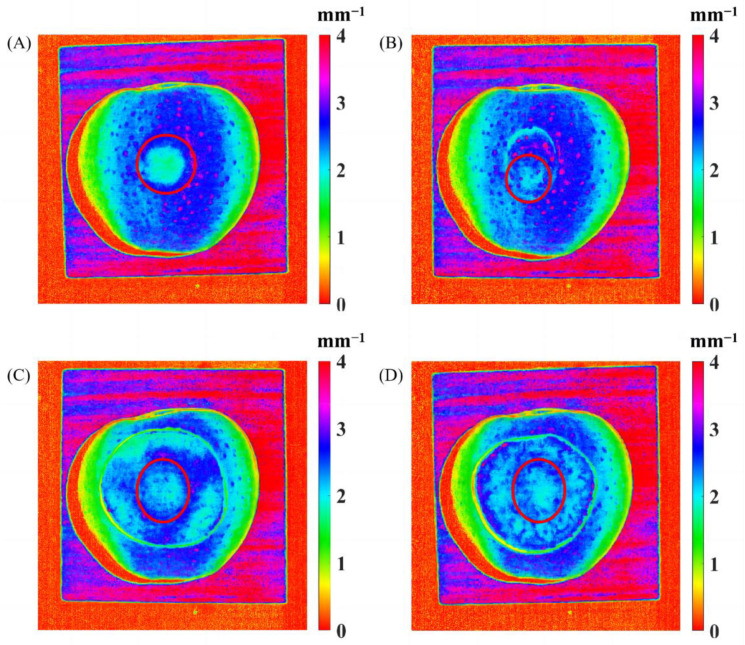
The reduced scattering coefficient mappings of the bruised apple covered without any slice (**A**), with a 1.2 mm thick slice with peel (**B**), and with a 0.8 mm thick (**C**) and 1.5 mm thick (**D**) slices without peel.

Figure 11 shows the reduced scattering coefficient mappings of the bruised apple covered with pre-prepared apple slices, with the bruised tissue marked in red circles. It was noticed that the bruised apple tissue without being covered by any slice, as shown in Figure 11A, was clearly observed from the reduced scattering coefficient mapping. Figure 11B shows that the bruised apple tissue covered with the 1.2 mm thick slice with peel could still be easily recognized, revealing that the spatially modulated light could completely penetrate through the apple slices with peel with the thickness of 0–1.2 mm. The 1.5 mm thick slice cover without peel (Figure 11D) provided more difficulty for apple bruise detection than the 0.8 mm thick slice (Figure 11C), but both of them could still be penetrated by the light. Through data analysis of all the mapping results with different-thickness slices, it was concluded that the apple slice without peel could be completely penetrated with the thickness range of 0–1.5 mm.

According to the report of Binzoni et al. [39], the number of photons that visit a given tissue voxel situated at a depth larger than 2 mm represents less than the 1% of the total number of photons reaching the corresponding detection pixel. They made the conclusion that the light penetration depth was no more than 2 mm, which confirmed our findings. In addition, Lu and Lu [21] reported that the maximum light penetration depth was no more than three sheets of white paper (or less than 400 μm). In our study, the apple slice, instead of white paper, was used as the cover for experiments, which was more scientific and reasonable for investigating the light penetration depth in apples. It should be pointed out that the results of light penetration depth obtained in this study are based on the apple sample, SFDI system configuration, and data processing method. Zhao et al. [16] reported that shortwave-infrared illumination could penetrate thicker biological tissue than visible light, because there is decreased optical scattering in the shortwave-infrared region compared to visible wavelengths. Hence, the hardware of the SFDI system, including the camera, the light source, and wavelength range, as well as the image processing algorithm, could be improved to increase light penetration depth in the future.

## 5. Conclusions

This paper presents theoretical and experimental analyses of light penetration depth in apple tissues using SFDI technology. The MC simulation coupled with the developed program indicated that the light penetration depth in apples was no more than 2.2 mm. The practical experiment on the USAF-1951 target covered with different-thickness apple slices demonstrated that the maximum light penetration depths were 1.8 mm with peel and 2.3 mm without peel, which were quite similar to the simulation results (0–2.2 mm). An example experiment on early-stage bruise detection was carried out to validate the light penetration depths in apples. The results showed that the bruised apple tissues covered with the slices with the thicknesses of 0–1.5 mm or 0–1.2 mm could be detected, depending on the presence of the apple peel or not. The maximum depth of an apple bruise should be about 1.2 mm beneath the surface peel, otherwise the SFDI technique cannot achieve accurate detection. In summary, SFDI can serve as a subsurface imaging technique for detecting early-stage bruises of thin-skinned fruits (<1.2 mm), such as apples. Improvement of system hardware and the data processing algorithm may provide potential for increasing the light penetration depth. Further work can be directed to explore the full potential of SFDI in bruise detection of other thin-skinned fruits and vegetables, such as pears, peaches, cucumbers, and potatoes.

## Figures and Tables

**Table 1:** Optical property parameters ($\mu_{a},{\mu^{\prime }}_{s}$) of ‘Golden Cream Delicious’ apple tissues measured by integrating sphere system at six different wavelengths.

Wavelength (nm)	$\mu_{a}\;({\mathrm{mm}}^{-1})$	${\mu^{\prime }}_{s}\;({\mathrm{mm}}^{-1})$
550	0.0302	1.286
600	0.0232	1.273
630	0.0218	1.259
675	0.0224	1.251
710	0.0222	1.256
730	0.0223	1.256

## Data Availability

The data underlying this article will be shared on reasonable request to the corresponding author.
